# Reasons for non-participation and dropout in a longitudinal study of an app-based support service among adult patients in a psychiatric outpatient setting during the COVID-19 pandemic

**DOI:** 10.3389/fpsyt.2025.1470554

**Published:** 2025-06-26

**Authors:** Luisa Kaufmann, Sabrina Baldofski, Konstanze Golsong, Elisabeth Kohls, Christine Rummel-Kluge

**Affiliations:** ^1^ Department of Psychiatry and Psychotherapy, Medical Faculty, Leipzig University, Leipzig, Germany; ^2^ Department of Psychiatry and Psychotherapy, University of Leipzig Medical Center, Leipzig, Germany

**Keywords:** non-participation, dropout, eHealth, app, mental health, mental disorder

## Abstract

**Background:**

Increasing non-participation in research studies and high dropout rates in research on mental health apps compromise interpretability and generalizability of results. Analyzing underlying reasons holds promise for improving future recruitment methods, study design, and app features.

**Objective:**

This study investigated reasons for non-participation and dropout among adult psychiatric outpatients in a study examining an app for self-reflection, daily structuring, relaxation, mindfulness, and psychoeducation in Germany during COVID-19 pandemic, as well as potential differences between dropouts and completers.

**Methods:**

Descriptive statistics on reasons for non-participation using an anonymous questionnaire and for dropout based on semi-structured telephone interviews were performed. Differences between dropouts and completers in sociodemographic, clinical, app-related, and daily mood data were analyzed.

**Results:**

Of *N* = 88 persons approached for potential study participation, *n* = 57 (64.8%) participated in the app study, while *n* = 31 (35.2%) declined. Of *n* = 31 non-participants, *n* = 29 (93.5%) indicated specific reasons. On average, *M* = 1.72 (*SD* = 1.03) reasons were provided per non-participant, with no motivation for regular app use (*n* = 7, 24.1%), no interest in using an app for the presented content (*n* = 6, 20.7%), and no time for app use (*n* = 6, 20.7%) being the most common. Of *n* = 57 study participants, *n* = 40 (70.2%) were completers and *n* = 17 (29.8%) were dropouts. On average, *M* = 2.82 (*SD* = 1.29) dropout reasons were provided per dropout, with too severe health complaints (*n* = 6, 35.3%), not individually suitable contents (*n* = 5, 29.4%), and lack of incentives to use the app (*n* = 5, 29.4%) being the most frequent. Dropouts and completers did not differ significantly in sociodemographic, clinical, and app-related variables (all *p* > .05). Dropouts reported their mood significantly less often than completers during the first five and seven days of the intervention period (all *p* < .001).

**Conclusions:**

This study provides exclusive insights into non-participation and dropout in an app study among adults with mental disorders. It identified personal motivation, app-related aspects, no interest in app-based offers, and personal health complaints as common reasons. Suggestions for improving future studies include focusing on incentives, app questionnaires, app installation, user needs analysis, and symptom severity. Early app engagement and adherence measurements (for example number of daily mood reports) may help identify potential dropouts earlier in future studies.

**Clinical Trial Registration:**

https://drks.de/search/de/trial/DRKS00027536, identifier DRKS00027536.

## 1 Introduction

Mental disorders are highly prevalent, affecting approximately one billion people globally (1) and ranking among the main causes of the global burden of disease (2). Anxiety disorders and depression are the leading two mental disorders worldwide (2), with rates increasing within the first year of the COVID-19 pandemic (3). In Germany, the COVID-19 pandemic also led to a worsening of depressive and anxiety symptoms, as well as a decline in subjective mental well-being (4). Mental disorders affect more than a quarter of the adult population in Germany every year and in up to half of cases there is a comorbidity of several mental disorders (5, 6). Together with the high direct and indirect costs associated with mental illness (1, 7), prevention and early, effective treatment are crucial. However, only 18.9% of those affected use health care services related to mental health in Germany annually, with many seeking professional help only years after symptom onset (8).

Known reasons for not starting or completing psychotherapy include transportation and time requirement, concerns about approaching a stranger with private issues or about stigmatization, treatment costs, and disorder-specific symptomatology, such as depressive symptoms, may also exacerbate these obstacles (9). Through cost-effectiveness, low-threshold access, flexibility in place and time, and anonymity, the implementation of e-mental health interventions may overcome these barriers and lead to improved access to mental health services (10). E-mental health comprises the application of information and communication technology for the enhancement and support of mental health care and mental health issues (11). Examples include virtual reality technologies, web-based interventions, and smartphone apps. Studies show the potential of apps in the area of mental disorders, especially depressive or anxiety disorders, as an add-on to existing therapies or as stand-alone self-management tools (12). Since the commencement of an act on digital health care in 2019, physicians and psychotherapists in Germany can now prescribe digital health applications, with costs reimbursed by health insurance companies (13), for example for the treatment of mental disorders (14).

Integrating online interventions, especially apps, into therapy is a logical step, as smartphone and internet use are now ubiquitous, and were reported by over 90% of patients in the Department of Psychiatry and Psychotherapy of the University of Leipzig (Germany) (15). Providing exclusive insight into the everyday life of patients, mobile technology-based services can monitor and support patients in real time in the form of ecological momentary assessment (EMA) (16) and intervention (EMI) (17). Studies suggest a potential in supporting health behavior using EMA-based EMIs via smartphone (18).

Research on smartphone apps has increased rapidly in recent years regarding their use in treating mental disorders, demonstrating positive effects when using intervention apps, especially for depressive and anxiety disorders (19). Achieving high participation rates in such studies is essential for accurate interpretation and generalization of research results, prevention of selection bias, and attainment of sufficient statistical power. As clinical research is dependent on voluntary study participation, the analysis of non-participation and dropout becomes highly relevant for improving recruitment and planning future studies on app-based support services.

Participation in research studies in general has decreased (20), non-participation is a known problem in health promotion studies (21), and high dropout rates have been shown in research on mental health apps (22). Considering these findings, it is very important to analyze reasons for non-participation and dropout, especially in clinical populations, such as patients with a mental disorder. According to previous studies, reasons for non-participation in mental health research include stigmatization, lack of trust, language barriers and transportation requirements (23, 24). Known reasons hindering the implementation and use of e-mental health tools comprise disorder-specific symptoms, lack of interest, rejection of technology in healthcare (25), and privacy concerns (26). Currently, data on reasons for non-participation and dropout in research on mental disorders, especially regarding app-based services in standard mental health care, remain limited.

Within a prospective longitudinal observational study examining an app-based support service in a psychiatric outpatient department in Germany during the COVID-19 pandemic (27), this study focused on the following four investigations. First, this study aimed to identify reasons for non-participation in the study using an anonymous questionnaire. Second, it aimed to assess reasons for dropout from the study based on semi-structured telephone interviews. Third, this study investigated potential differences between dropouts and completers in sociodemographic information, clinical and app-related parameters. Finally, it explored potential differences between dropouts and completers in daily mood data assessed via EMA within the app. Based on findings from previous literature that dropout or lower study adherence can be associated with feeling too ill (28), severe depressive disorders (29), and technical problems with app use (30, 31), the study hypothesized that dropouts would be more affected by depressive symptoms, suicidal ideation, psychological distress triggered by the COVID-19 pandemic, and would report more problems with the app compared to completers.

## 2 Materials and methods

### 2.1 Participants and procedure

Data were collected as part of a prospective longitudinal observational study examining an app-based support service among a sample of adult patients in the psychiatric outpatient department of Leipzig University Hospital (Germany) between February and April 2022 (27). As a feasibility study, the intervention was conducted in a single-arm design.

The study took place when numerous governmental restrictions were still enforced in response to the COVID-19 pandemic. For example, outpatient face-to-face group therapies could only be conducted with a limited number of participants. The study was designed to serve as an additional support service to existing outpatient treatments, without interfering with or replacing existing treatments and therapies. Regardless of their specific diagnosis of a mental disorder, all *N* = 88 patients who were currently receiving outpatient treatment at the psychiatric outpatient department of Leipzig University Hospital and who had an appointment with their psychotherapist during the recruitment period between February 21, 2022 and March 14, 2022 were informed about the study in a face-to-face meeting with study staff before or after their appointment with their psychotherapist. They were able to see some of the app´s content and also received an information sheet about the study, that included screenshots of the app.

The inclusion criteria were current treatment in the psychiatric outpatient department of Leipzig University Hospital, an age of 18 years or older, internet access, possession of a smartphone that supported downloading the app, adequate German language skills, sufficient vision and reading ability, capacity to consent, and the ability to answer the questionnaires independently. The exclusion criteria were pregnancy and breastfeeding. The ethics committee of the Medical Faculty of the University of Leipzig granted ethical approval for this study (558/21-ek, 12/20/2021), and the study was registered in the German Clinical Trials Register (DRKS00027536).

Of the *N* = 88 patients invited to participate in the study, *n* = 31 (35.2%) declined and did not provide written informed consent. These patients were asked to specify their reasons for non-participation by completing an anonymous paper-and-pencil questionnaire. The respondents were informed that the questionnaire was completely anonymous and that no conclusions could be drawn about the individual person. It was also explained that the answer given in the questionnaire would have no consequence on their current treatment in the psychiatric outpatient department. In total, 93.5% (*n* = 29) of the non-participants indicated specific reasons for their non-participation.

Of all *N* = 88 patients approached for potential study participation, *n* = 57 (64.8%) patients gave their written informed consent for study participation. They subsequently received both the installation instructions and a manual for the app, and installed the app on their own smartphones together with the study staff. After that, the baseline questionnaire (T0, pre-intervention) was activated within the app. After completing the baseline questionnaire, the intervention period started and all the data generated in the app was recorded (T1, during intervention). Within the first week study participants who had consented to a follow-up call at the time of study inclusion were contacted by study staff to clarify any problems or questions (T1). At the end of the four-week intervention period, the final questionnaires (T2, post-intervention) were activated within the app. Participants were reminded to complete them via push message on their smartphone and via an additional phone call.

All participants who provided written informed consent for study participation and completed the baseline questionnaire (T0), but either missed using the app for one entire week during the four-week intervention period or notified study staff by telephone or e-mail that they did not want to participate in the study anymore were defined as dropouts (*n* = 17, 29.8%). If participants met the definition for a dropout, they were contacted via telephone and asked about the reasons for the dropout using a semi-structured interview guide. Only linked to the study ID, all interview content was kept confidential so that no conclusions could be drawn about the natural person, and dropouts were informed that the given answers had no impact on the ongoing treatment in the psychiatric outpatient department. The interviews were performed during the study period immediately after dropout to minimize recall bias. Semi-structured telephone interviews were conducted with all *n* = 17 (100.0%) dropouts to identify reasons for dropout from the study.

All participants who used the app regularly during the four-week intervention period were defined as completers (*n* = 40, 70.2%). Regular app use was met if at least one of the three app sections was used at least once a week, for example if a daily questionnaire was completed in full or an exercise module was used within the applet, and data was thus generated within one of the three app sections. Among completers, it was demonstrated that the app-based support service was feasible, accepted and associated with high user satisfaction (27).

### 2.2 App-based support service

During the design phase of the app-based support service in December 2021, two female patients currently receiving treatment in the psychiatric outpatient department of Leipzig University Hospital participated in the development process of the app and the planning of the study as part of an online focus group. Both patients volunteered for the focus group and did not participate in the intervention phase of the study afterwards [for details please see (27)].

Using an internet platform called m-Path developed by KU Leuven (Belgium) to conduct EMA and EMI using smartphones (32), the authors created an unguided support app that study participants could use regularly and independently in their daily lives during the four-week intervention period. The app offered low-threshold questionnaires and exercise modules for self-reflection, daily structuring, relaxation, mindfulness, and psychoeducation, and was designed for participants with different diagnoses of mental disorders. Data safety, privacy, and anonymity in handling study participant data were ensured by the fact that communication between the smartphone and the server is end-to-end encrypted and study participants did not need to enter sensitive contact information like e-mail address, phone number, or name when registering in the app (32).

The app included three major sections [for details please see (27)]. Section one consisted of three short daily questionnaires that regularly assessed, inter alia, mood in the sense of an EMA. These questionnaires were unlocked within the app in the morning, at noon, and in the evening, and could be answered once within a six-hour interval. Push messages on the smartphone reminded participants to complete these daily questionnaires. The reported mood data were displayed as a graph within the app, allowing study participants to monitor their mood trend during the intervention period. The second section comprised an activity button on the app’s home screen which asked participants to record both scheduled and completed activities for that day or suggested potential activities to support daily structuring and motivation for activities. The study participants could choose from predefined answer options for scheduled activities (appointment with a doctor or psychologist, appointment in group therapy, leisure activity, official appointment, appointment at work) or enter their own answers. These custom responses were stored as additional answer options the next time the activity button was opened. The third section was an applet titled “Exercises for you - be kind to your mind”, which was specifically designed for the purpose of this study. The applet provided seven different exercise modules focusing on relaxation, mindfulness, and psychoeducation. The app included an award system. Awards were given for completed activities entered via the activity button and for completing module contents within the applet in the sense of positive reinforcement.

### 2.3 Measures

Except for the reasons for non-participation and dropout, all data were recorded via the app. Reasons for non-participation were determined using an anonymous questionnaire, while reasons for dropout were determined based on semi-structured telephone interviews.

#### 2.3.1 Anonymous questionnaire on reasons for non-participation

An anonymous paper-and-pencil questionnaire was used to assess reasons for non-participation among all respondents who declined to participate in the study. During the development of this questionnaire for the purpose of this study, potential reasons for non-participation or non-completion commonly described in other studies on web-based interventions or in patients with mental disorders were included (33, 34). The items were pretested within the working group for content, relevance, comprehensibility, and duplications in the answer options and were adapted if necessary. The final one-page questionnaire contained the following thirteen potential reasons for non-participation: “1 = No (further) study participation wanted”, “2 = Lack of technical requirements”, “3 = Health complaints too severe”, “4 = No interest in an app-based support service”, “5 = Not liking the content presented”, “6 = No additional therapy support needed”, “7 = Already using an app-based support service”, “8 = No interest in using an app for the presented content”, “9 = No motivation for regular app use”, “10 = No time for app use”, “11 = Disliking the procedure of the study”, “12 = Not wanting personal data to be stored”, “13 = Lack of financial compensation”. The presence of each reason could be indicated by the respondent, and selection of multiple reasons was possible.

In addition, a free-text field allowed respondent to add further reasons for non-participation or explanations for a given answer. The respondents also had the option to refuse to state the reason for non-participation by choosing the additional item “I do not want to indicate a reason”. All selected reasons in the non-participation questionnaire as well as the additional reasons given in the free-text field were analyzed separately and were then grouped into the following four main categories defined by study staff after reviewing all responses: “1 = App-related reasons”, “2 = Individual reasons”, “3 = Organizational reasons”, and “4 = Reasons related to study participation in general”. The questionnaire was deliberately kept short and anonymous to obtain spontaneous, honest responses from non-participants. This approach avoided overwhelming them with too many items or a long processing duration for this questionnaire, and to minimize the number of non-participants who chose not to provide a reason for their non-participation.

#### 2.3.2 Semi-structured interview guide on reasons for dropout

To determine reasons for dropout, a telephone interview was conducted with all participants who met the definition of a dropout. For the purpose of this study, a semi-structured interview guide was developed by study staff to ensure a standardized framework. Using suggestions made by Helfferich (35) regarding the development of a guideline and conducting guideline-based interviews, the interview guide consisted of main questions designed to encourage the interviewee to provide a free narrative about the topic. It also included several follow-up content questions that could be asked if these aspects had not yet been addressed in the response to the main questions to ensure a structure. The course of the interview could deviate from both the order and the exact wording of the questions in the interview guide and could be modified to accommodate each partner’s individual weighting of the various interview subjects and their narrative flow. Arising of additional questions was allowed, and at the end of the interview, respondents were asked for further additions to the topics discussed.

The interview guide contained eight main questions. The topics covered the motivation for participating in the study of the app-based support service, the usage behavior regarding the three main contents of the app, an assessment of which specific app contents were liked and disliked, feedback on the app format, the change in usage behavior during the intervention period, the reasons to stop using the app, and experiences with other app-based support services.

Two PhD students (LK, KG) conducted the interviews and were in regular exchange to standardize the interview procedure to ensure the quality of the interviews. All interviews lasted between 5–30 minutes. During the interview, the topics discussed, and the answers given were written down in a short protocol in pseudonymized form and the reasons for dropout were extracted. Some patients gave multiple reasons for dropout. After reviewing all short protocols of the telephone interviews and reasons given for dropout extracted from them, the reasons for dropout could be grouped into the following three different main categories defined by study staff: “1 = App-related reasons”, “2 = Individual reasons”, and “3 = Use of alternative offers for relaxation”. Emerging disagreements on categorization of specific given reasons for dropout were discussed between study staff until consensus was reached.

#### 2.3.3 Sociodemographic variables, diagnosis, pandemic-related and technical information

The baseline survey (T0) contained information on sociodemographic data (age, gender, relationship status, living situation, having children, children living in the household, employment status). In addition, the main diagnosis was recorded according to the “International Statistical Classification of Diseases and Related Health Problems, 10th Revision, German Modification” (ICD-10-GM) (36), for which the study participants were receiving treatment at the psychiatric outpatient department of Leipzig University Hospital on the basis of their therapists’ medical records. The following diagnosis codes from Chapter V “Mental and behavioral disorders” of the ICD-10-GM (36) were represented in the study: paranoid schizophrenia (F20.0), affective disorders (F31.0, F31.6, F31.7, F31.8, F32.1, F32.2, F33.1, F33.2, F33.4, F34.1), neurotic, stress-related and somatoform disorders (F40.01, F40.1, F41.0, F41.1, F42.0, F42.2, F43.1, F45.1, F45.2), severe mental and behavioral disorders associated with the puerperium, not elsewhere classified (F53.1), emotionally unstable personality disorder, borderline type (F60.31), and disturbance of activity and attention (F90.0). Further, the extent of perceived psychological distress triggered by the COVID-19 pandemic was rated on a 4-point Likert scale from “0 = not at all” to “3 = very much”. Moreover, a dichotomous answer format (yes/no) was used to record whether any problems with the app had occurred within the first week after study inclusion based on a follow-up call by study staff (T1).

#### 2.3.4 Quality of life

Measuring the subjectively perceived quality of life of the study participants, the abbreviated World Health Organization Quality of Life Assessment (WHOQOL-BREF) (37, 38) was assessed at T0 and T2. It can be used regardless of a specific disease (39) and includes 26 items (37, 39) covering the past 2 weeks, using a 5-point Likert scale ranging from “1 = very poor/very dissatisfied/not at all/never” to “5 = very good/very satisfied/an extreme amount/extremely/completely/always”. The items are assigned to the four health domains of physical health, psychological health, social relationships, and environment (37). A separate domain score is calculated for each domain (37), which is converted to a scale from “0 = worst possible health status” to “100 = best possible health status”. The questionnaire is widely used in research and has a high level of validity and very good psychometric properties (37, 39).

#### 2.3.5 Depressive symptoms and suicidal ideation

Depressive symptoms were assessed at T0 and T2 using the Patient Health Questionnaire-9 (PHQ-9) (40). The PHQ-9 is an internationally used 9-item questionnaire assessing depressive symptoms within the past 2 weeks according to DSM-IV criteria for major depression on a 4-point Likert scale ranging from “0 = not at all” to “3 = nearly every day” (40), resulting in a total sum score from 0 to 27. Higher scores indicate higher depressive symptomatology. Further, item 9 of the PHQ-9 assessing suicidal ideation was analyzed separately. It was recorded whether suicidal ideation (in case of a given answer ≥ 1 on item 9) was present (yes/no) and how often suicidal ideation occurred in the past two weeks on a 4-point Likert scale (“0 = not at all”, “1 = several days”, “2 = more than half the days”, “3 = nearly every day”).

Study staff were able to view the responses given in the app in the online dashboard. If the study participant gave an answer ≥ 1, a standard operating procedure for suicidality established in the psychiatric outpatient department of Leipzig University Hospital was conducted. This included an automatic alert on the smartphone screen of the participant with a request to report immediately to the psychiatric outpatient department or to call the national emergency number. In addition, the situation was explored by the study staff in a face-to-face conversation at T0 or by a phone call at T2 with the study participant and the suicide risk was assessed. Likewise, a report was made to the treating physician or psychotherapist.

According to a recent meta-analysis of individual participant data (IPDMA), the combined sensitivity and specificity was highest for a PHQ-9 cut-off score ≥ 10 compared with semi-structured reference standards to detect clinically significant depressive symptomatology (41). The PHQ-9 has been shown to have a high level of internal reliability and test-retest reliability (40). Studies have confirmed the high internal consistency as well as test-retest reliability of the PHQ-9 using a smartphone (42) and an unaffected reliability of the PHQ-9 when the paper version is converted to a smartphone version (43).

#### 2.3.6 Treatment credibility and expectancy

To assess treatment credibility and expectancy, the credibility/expectancy questionnaire (CEQ) (44) was administered at T0 and T2. The questionnaire was translated into German by the working group using a back-translation method (45). Its wording was adapted to the intervention via smartphone. The CEQ is divided into a first part (thinking-related) comprising four items and a second part (feeling-related) including two items (44). Four items are rated on a 9-point Likert scale from “1 = not at all/not at all logical/useful/confident” to “9 = very much/logical/useful/confident” and two items are assessed on an 11-point Likert scale from “0%” to “100%” (44). Before calculating treatment credibility and expectancy, all items were standardized (44). A credibility factor was then calculated by summing the standardized scores of the first three items of the first part of the CEQ (44). Adding the standardized scores of the remaining items of both parts of the CEQ results in an expectancy factor (44). The sum scores of both factors ranged from 3 to 27, whereby higher values correspond to a higher treatment credibility and expectancy, respectively. A high level of test-retest reliability and good internal consistency were shown by the CEQ (44).

#### 2.3.7 Daily mood data of dropouts and completers assessed via EMA

To represent the mood data of the sample of dropouts and completers, the mood of all participants was assessed in the morning, at noon, and in the evening via three automated daily questionnaires during the four-week intervention period (T1) of the app-based support service in the sense of an EMA. If a questionnaire was not answered within a six-hour interval, the mood data for that time interval was missing. In addition, participants’ mood was recorded before and after completing each exercise module in the applet (T1). Mood level was rated on a visual smiley scale from 0 to 100.

All persons in the dropout group from whom mood data were available (*n* = 14, 82.4%) reported mood data within the first twelve days of the four-week intervention period. After these twelve days, mood data were only reported by less than half of these persons (*n* = 6, 42.9%) in the remaining days (day 13 to 28) of the four-week intervention period. For this reason, only mood data collected within the first twelve days of the four-week intervention period were considered for statistical analysis.

For each day (day 1 to day 12), all available mood data of a participant within one day were averaged and a daily mean score of the mood level was calculated. From these daily mean mood scores of the individual participants, an overall daily mean mood score was calculated for the dropout and completer group, respectively, and the mood level trend was displayed graphically.

Additionally, the number of mood reports provided by each participant was determined per day (day 1 to day 12) and an overall daily mean of the number of mood reports was calculated for the dropout and completer group, respectively. The overall mean values of the number of mood reports per day for each of the two groups were presented graphically.

The definition of a dropout in our study meant that the app was not used for one entire week during the four-week intervention period. Based on this, it was of particular interest to investigate whether the number of daily mood reports differed between dropouts and completers within the first seven days of the four-week intervention period, and whether it differed even earlier, within the first five days. The latter is important as it may help to identify potential dropouts before they reach the dropout time point defined in the study. This means that potential dropouts can be contacted at an early stage and a dropout can possibly be prevented. Accordingly, the total number of daily mood reports during the first seven days as well as during the first five days of the app intervention period was calculated for all participants in the dropout and completer group, respectively, and compared between the groups.

### 2.4 Statistical analysis

First, descriptive statistics were performed on reasons given for non-participation and second on reasons for dropout. Third, descriptive statistics in the sample of dropouts and the sample of completers, respectively, on sociodemographic information, main diagnosis, WHOQOL-BREF, PHQ-9, the presence as well as frequency of suicidal ideation (item 9 of PHQ-9), extent of perceived psychological distress triggered by the COVID-19 pandemic, CEQ, existence of problems with the app within the first week after study inclusion, and fourth, on daily mood data were reported and group differences were analyzed.

To examine differences between dropouts and completers in sociodemographic information, clinical and app-related parameters as well as mood data, *χ^2^* tests were used for all categorical dependent variables (gender, relationship status, living situation, having children, children living in the household, employment status, main diagnosis, presence of suicidal ideation, existence of problems with the app within the first week after study inclusion). An important note concerns the *χ^2^* tests of the variables gender, relationship status, living situation, employment status, and main diagnosis, in which some cell frequencies were below five, which impedes a correct interpretation of these results. In these cases, the exact significance (two-sided) for the *χ^2^* tests was reported. Effect sizes for *χ^2^* tests were interpreted according to the recommendations of Cohen using φ coefficients for 2x2 crosstabulations and Cramérs *V* for crosstabulations larger than 2x2 (46).

Differences in continuous dependent variables (age, frequency of suicidal ideation, perceived psychological distress triggered by the COVID-19 pandemic, number of daily mood reports) with non-normal distribution (as indicated by Shapiro-Wilks test, *p* <.05) were evaluated using Mann-Whitney *U* tests. The effect size for Mann-Whitney *U* tests was *r* (46). Independent *t* tests for all continuous dependent variables (WHOQOL-BREF, PHQ-9, CEQ) with normal distribution (as indicated by Shapiro-Wilks test, *p* >.05) were performed. For estimating effect sizes for independent *t* tests, Cohen *d* was used (46). IBM SPSS Statistics, version 28.0.1.1, was used to conduct the statistical analyses using a two-tailed α = .05. Only valid values were reported in the descriptive statistics.

## 3 Results

### 3.1 Reasons for non-participation in the study

The results from the non-participation questionnaires are presented in **Table 1**. Of the total number of *n* = 31 non-participants, *n* = 29 (93.5%) indicated specific reasons for their non-participation, while *n* = 2 (6.5%) refused to state the reason for their non-participation by choosing the item “I do not want to indicate a reason”. In relation to the total number of specific reasons given for non-participation, a mean of *M* = 1.72 (*SD* = 1.03; range 1–5) reasons for non-participation were given per person. Of the thirteen potential reasons for non-participation, ten different reasons were indicated (see **Table 1**). The most frequently indicated reasons were “No motivation for regular app use” (*n* = 7, 24.1%), “No interest in using an app for the presented content” (*n* = 6, 20.7%), and “No time for app use” (*n* = 6, 20.7%). The answer options “Not liking the content presented”, “Disliking the procedure of the study” and “Lack of financial compensation” were not indicated by any person.

**Table 1:** Reasons for non-participation (*n* = 29).

Reasons for non-participation, *n* (%)	Non-participants (*n* = 29)
App-related reasons	12 (41.4)
No motivation for regular app use	7 (24.1)
No interest in using an app for the presented content	6 (20.7)
No interest in an app-based support service	2 (6.9)
Already using an app-based support service	1 (3.4)
*Wish for additional app content*	1 (3.4)
*Feeling not taken seriously by the awards in the app*	1 (3.4)
*Feeling overwhelmed and anxious about app use*	1 (3.4)
Individual reasons	15 (51.7)
No time for app use	6 (20.7)
*No time for app installation*	5 (17.2)
Health complaints too severe	3 (10.3)
No additional therapy support needed	1 (3.4)
*Preference for an offline offering*	1 (3.4)
*No experience in smartphone use*	1 (3.4)
Organizational reasons	8 (27.6)
Inclusion criteria not met (lack of technical requirements, *pregnancy, no German language skills)*	7 (24.1)
*Error during app installation*	1 (3.4)
Reasons related to study participation in general	5 (17.2)
Refusal to store personal data	3 (10.3)
No (further) study participation wanted	2 (6.9)
*Participation in another study*	1 (3.4)

Indication of multiple reasons was possible.

Bold: main categories of reasons for non-participation.

Not italic: response options for reasons for non-participation indicated in the non-participation questionnaire.

Italic: reasons for non-participation reported in the free-text field of the non-participation questionnaire.

Further, a total of ten different reasons for non-participation were reported in the free-text field (see **Table 1**). The most frequent reason reported in the free-text field was “No time for app installation” (*n* = 5, 17.2%).

The resulting twenty different reasons given for non-participation, as combined from the ten potential reasons indicated in the questionnaire and the ten additional reasons given in the free-text field answers, could be grouped into four distinct main categories (bold in **Table 1**). Of all individuals stating reasons for their non-participation, the majority reported reasons in the main category of “individual reasons” (*n* = 15, 51.7%), followed by the main category of “app-related reasons” (*n* = 12, 41.4%), and the main category of “organizational reasons” (*n* = 8, 27.6%). The fewest non-participants indicated reasons in the main category of “reasons related to study participation in general” (*n* = 5, 17.2%).

### 3.2 Reasons for dropout from the study

**Table 2** shows all reported reasons for dropout extracted from the short protocols of the telephone interviews grouped into three defined main categories (bold in **Table 2**). Concerning the total number of reasons given for dropout, a mean of *M* = 2.82 (*SD* = 1.29; range 1–5) reasons for dropout were given per person in the sample of dropouts (*n* = 17, 29.8%).

**Table 2:** Patient-reported reasons for dropout (*n* = 17).

Reasons for dropout, *n* (%)	Dropouts (*n* = 17)
**App-related reasons**	14 (82.4)
Contents are not individually suitable	5 (29.4)
Lack of incentives to use the app	5 (29.4)
Push messages not noticed	4 (23.5)
Push messages are annoying	3 (17.6)
Layout not liked	3 (17.6)
Too few variations in the questions within the app	1 (5.9)
Too few functions	1 (5.9)
Contents not liked	1 (5.9)
Predefined activities in the activity button are too problem-oriented and are perceived as negative	1 (5.9)
App icon too inconspicuous	1 (5.9)
No reminder for the activity button and relaxation modules	1 (5.9)
Individual reasons	12 (70.6)
Health complaints too severe	6 (35.3)
No time to use the app	4 (23.5)
No motivation to use the app (regularly)	3 (17.6)
Feeling of chronic mental overload	1 (5.9)
Too much stress	1 (5.9)
Smartphone change	1 (5.9)
Use of alternative offers for relaxation	5 (29.4)
Use of other smartphone apps	2 (11.8)
Use of face-to-face offerings	2 (11.8)
Use of offerings on YouTube	1 (5.9)
Use of an own program on smartphone, without internet	1 (5.9)

Indication of multiple reasons was possible.

Bold: main categories of reasons for dropout.

Of the total sample of dropouts, most individuals (*n* = 14, 82.4%) cited app-related reasons for their dropout. Most frequent reasons for dropout in this main category were “Contents are not individually suitable” (*n* = 5, 29.4%), “Lack of incentives to use the app” (*n* = 5, 29.4%) and “Push messages not noticed” (*n* = 4, 23.5%). In the main category “Individual reasons”, *n* = 12 (70.6%) individuals of the *n* = 17 dropouts gave reasons for their dropout. Most common reasons for dropout in this main category were “Health complaints too severe” (*n* = 6, 35.3%) and “No time to use the app” (*n* = 4, 23.5%). The fewest individuals (*n* = 5, 29.4%) cited reasons for their dropout in the category of “Use of alternative offers for relaxation”.

### 3.3 Sample characteristics and group differences between dropouts and completers

Of all study participants (*n* = 57, 64.8%), the sample of dropouts comprised 29.8% (*n* = 17) of the participants with a proportion of 64.7% (*n* = 11) female participants and a mean age of *M* = 33.88 years (*SD* = 11.38; range 18–59 years; see **Table 3**). The sample of completers included 70.2% (*n* = 40) of the participants consisting of 77.5% (*n* = 31) female participants and a mean age of *M* = 38.63 years (*SD* = 14.27; range 19–68 years; see **Table 3**). Most participants in both groups were single, lived with others, and had no children living in the household (see **Table 3**). More than one-third of the participants in the dropout sample were currently treated for affective disorders, while the largest proportion of participants in the completer sample currently received treatment for neurotic, stress-related, and somatoform disorders (see **Table 3**).

**Table 3:** Sample characteristics and group differences between dropouts (*n* = 17) and completers (*n* = 40).

Variable	Dropouts (*n* = 17)	Completers (*n* = 40)	Test	*p* value	Effect size
Age, *M* (*SD*)	33.88 (11.38)	38.63 (14.27)	*U* = 280.50	0.299	*r* = 0.14
Female gender, *n* (%)	11 (64.7)	31 (77.5)	*χ^2^* ^1^ = 1.01	0.341	φ = –0.13
**Relationship status, *n* (%)**			*χ^2^* ^3^ = 0.91	0.833	*V* = 0.13
Married	3 (20.0)	12 (30.0)			
Divorced	1 (6.7)	3 (7.5)			
In relationship	5 (33.3)	9 (22.5)			
Single	6 (40.0)	16 (40.0)			
Living situation together with others, *n* (%)	12 (80.0)	27 (67.5)	*χ^2^* ^1^ = 0.83	0.510	φ = 0.12
Having children, *n* (%)	7 (46.7)	21 (52.5)	*χ^2^* ^1^ = 0.15	0.700	φ = –0.05
Children living in the household, *n* (%)	6 (40.0)	14 (35.0)	*χ^2^* ^1^ = 0.12	0.731	φ = 0.05
**Employment status, *n* (%)**			*χ^2^* ^4^ = 2.92	0.622	*V* = 0.23
Employed	5 (33.3)	12 (30.0)			
Unemployed	1 (6.7)	2 (5.0)			
Retired or unable to work	2 (13.3)	13 (32.5)			
In school/training or in study	6 (40.0)	9 (22.5)			
On parental leave or housewife/houseman	1 (6.7)	4 (10.0)			
**Main diagnosis (ICD-10-GM), *n* (%)**			*χ^2^* ^5^ = 5.77	0.341	*V* = 0.32
Paranoid schizophrenia	1 (5.9)	3 (7.5)			
Affective disorders	6 (35.3)	13 (32.5)			
Neurotic, stress-related and somatoform disorders	4 (23.5)	15 (37.5)			
Severe mental and behavioral disorders associated with the puerperium, not elsewhere classified	0 (0.0)	1 (2.5)			
Emotionally unstable personality disorder, borderline type	1 (5.9)	5 (12.5)			
Disturbance of activity and attention	5 (29.4)	3 (7.5)			
**Quality of life (WHOQOL-BREF), *M* (*SD*)**					
Physical health	50.24 (14.25)	50.98 (17.74)	*t*_53_ = 0.15	0.885	*d* = 0.04
Psychological health	37.22 (14.56)	42.40 (17.40)	*t*_53_ = 1.02	0.311	*d* = 0.31
Social relationships	45.56 (22.90)	52.08 (20.48)	*t*_53_ = 1.02	0.313	*d* = 0.31
Environment	56.67 (18.56)	65.00 (15.84)	*t*_53_ = 1.66	0.103	*d* = 0.50
Depressive symptoms (PHQ- 9), *M* (*SD*)	13.60 (5.96)	13.58 (5.77)	*t*_53_ = –0.01	0.989	*d* = –0.00
Presence of suicidal ideation, *n* (%)	5 (29.4)	15 (37.5)	*χ^2^* ^1^ = 0.34	0.558	φ = –0.08
Frequency of suicidal ideation, *M* (*SD*)	1.80 (0.84)	1.93 (0.80)	*U* = 34.00	0.860	*r* = 0.07
Perceived psychological distress triggered by COVID-19 pandemic, *M* (*SD*)	1.80 (0.94)	2.15 (0.74)	*U* = 237.00	0.204	*r* = 0.17
**Treatment credibility and expectancy (CEQ), *M* (*SD*)**					
Credibility factor	16.80 (4.33)	18.25 (3.48)	*t*_53_ = 1.29	0.204	*d* = 0.39
Expectancy factor	13.27 (3.56)	14.55 (4.66)	*t*_53_ = 0.96	0.339	*d* = 0.29
Problems with the app in the first week after study inclusion, *n* (%)	0 (0.0)	0 (0.0)	N/A	N/A	N/A

Calculation of % from valid cases.

ICD-10-GM, International Statistical Classification of Diseases and Related HealthProblems, 10^th^ Revision, German Modification.

WHOQOL-BREF, abbreviated World Health Organization Quality of Life Assessment.

PHQ-9, Patient Health Questionnaire-9.

CEQ, Credibility/Expectancy Questionnaire.

N/A, not applicable.

When analyzing group differences between dropouts and completers no statistically significant differences were detected for any of the sociodemographic, clinical, and app-related variables (all *p* >.05; see **Table 3**). The effect sizes indicated no to medium effects, with predominantly small or weak effects (see **Table 3**).

### 3.4 Daily mood data of dropouts and completers assessed via EMA

Within the dropout group, mood data were available from *n* = 14 (82.4%) of the *n* = 17 dropouts within the first twelve days of the app intervention period. Of the remaining *n* = 3 (17.6%) persons of the *n* = 17 dropouts, no mood data were available because they had deleted all their data entered within the app by themselves (*n* = 2, 11.8%) or had only answered the baseline questionnaire (*n* = 1, 5.9%). Of the *n* = 14 dropouts with available mood data, a mean of *M* = 6.33 (*SD* = 2.64) participants per day provided mood data, with the number of individuals providing daily mood data varying between *n* = 3 (21.4%) and *n* = 11 (78.6%) individuals.

Within the completer group, data of *n* = 38 (95.0%) completers with complete data sets of the total *n* = 40 completers were used for calculating the mood data. Of these *n* = 38 completers with complete data sets, a mean of *M* = 34.08 (*SD* = 6.75) participants per day reported mood data, while the number of individuals providing daily mood data ranged from *n* = 13 (34.2%) to *n* = 38 (100.0%).

**Figure 1:**
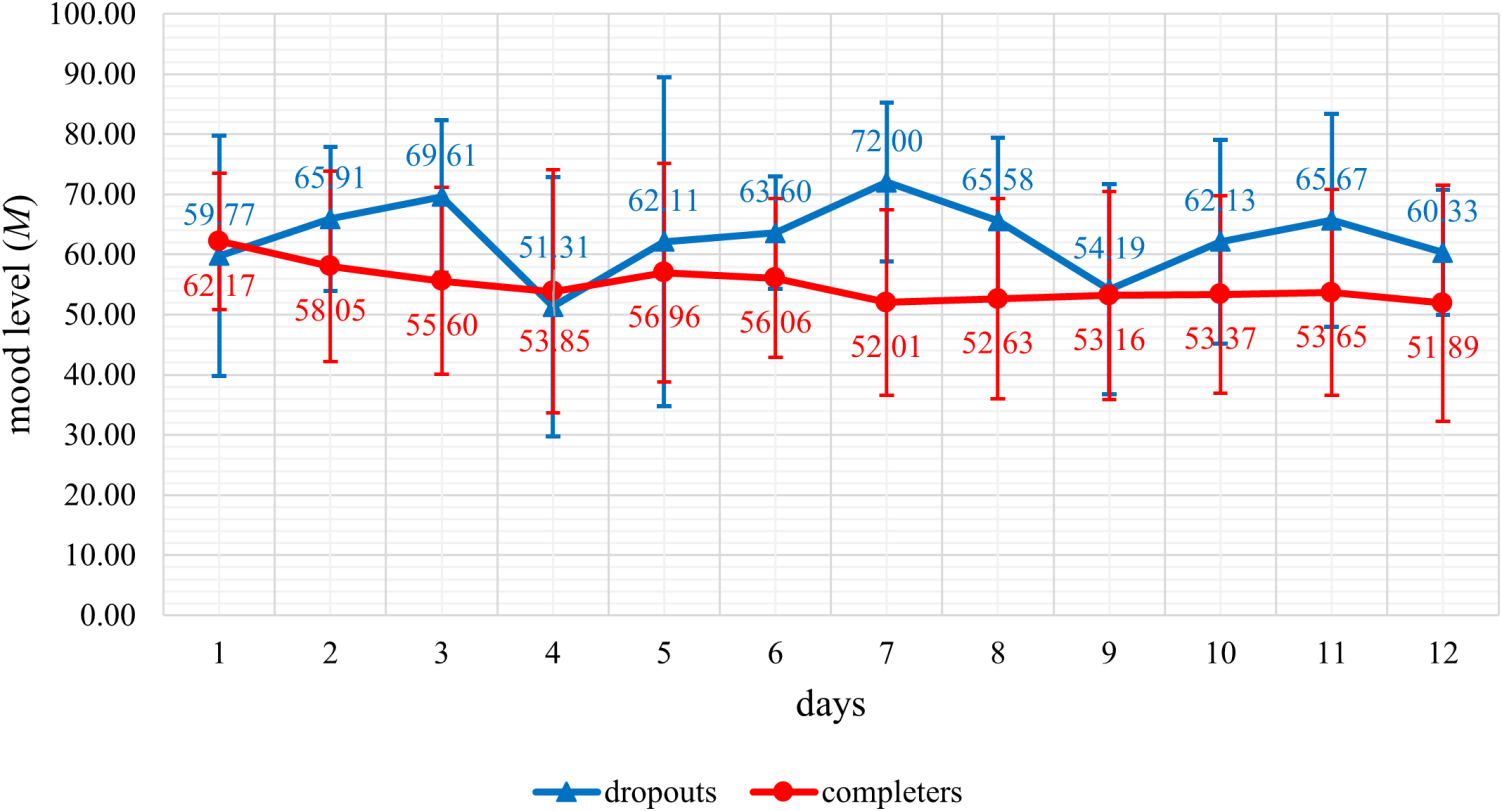
Representation of the daily mean mood levels of dropouts (*n* = 14) and completers (*n* = 38) during the first twelve days of the app intervention period.

**Figure 2:**
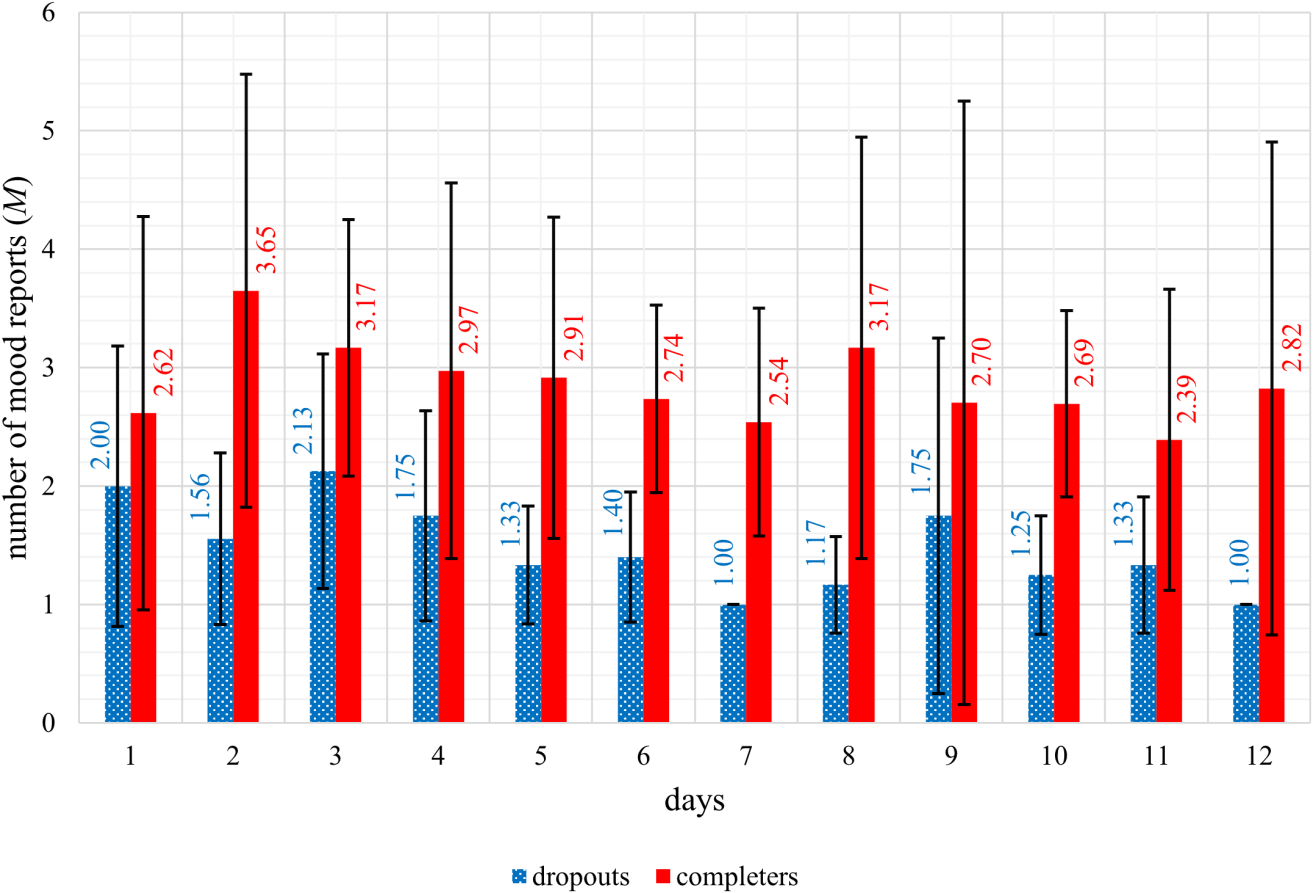
Representation of the mean number of mood reports of dropouts (*n* = 14) and completers (*n* = 38) per day during the first twelve days of the app intervention period.

The trend in mean mood levels (see **Figure 1**) and the mean number of mood reports per day (see **Figure 2**) were displayed graphically. The graphic representation shows a better mood trend in the dropout group of the mean mood levels, and a lower mean number of mood reports in the dropout group compared descriptively to the completers. When analyzing group differences between dropouts and completers in the number of daily mood reports statistically significant differences with strong effect sizes were detected for the number of daily mood reports during the first seven days (*U* = 25.50; *p* <.001; *r* = 0.67) as well as during the first five days (*U* = 48.00; *p* <.001; *r* = 0.61) of the app intervention period. Dropouts reported their mood significantly less often than completers during the first seven days as well as during the first five days of the app intervention period.

## 4 Discussion

### 4.1 Principal findings

The aim of this study was to provide insights into reasons for non-participation in a study examining an app for adult outpatients with a mental disorder using an anonymous questionnaire. Additionally, reasons for dropout were assessed through semi-structured telephone interviews. This study also aimed to determine potential differences between dropouts and completers in sociodemographic information, clinical and app-related parameters. In addition, daily mood data of dropouts and completers, assessed via EMA within the app, were presented, and group differences between dropouts and completers in the number of daily mood reports were analyzed.

Descriptive statistics of the non-participation questionnaire items and free-text responses indicate an average of two reasons for non-participation per person. The most common reasons cited were “No motivation for regular app use”, “No interest in using an app for the presented content”, “No time for app use”, and “No time for app installation”. On average, three reasons for dropout were given per person, with the most frequent being “Health complaints too severe”, “Contents are not individually suitable”, and “Lack of incentives to use the app”. Analysis of group differences between dropouts and completers showed significantly more daily mood reports among completers than dropouts during the first seven days as well as during the first five days of the app intervention period, with strong effect sizes.

The results provide a better understanding of why adult patients with mental disorders do not participate in a study investigating an app or drop out prematurely. While 21.7% of persons in the non-participation group and the dropout group combined indicated no motivation to use the app regularly, an equal proportion in both groups together reported a lack of time to use the app. Additionally, 29.4% of dropouts lacked incentives to use the app. These findings highlight the importance of personal motivation as a key factor influencing non-participation and dropout. To enhance personal motivation, more incentives to use the app should be created, for example in the form of personalized feedback (30, 31, 47). One option would be to integrate the app as an add-on to ongoing therapy, allowing app data to be discussed and incorporated into therapy sessions. Another option is by adding gamification elements (30, 31, 47) or push notifications to encourage motivation (30, 31, 47). More push notifications can also improve app engagement, considering that “Push messages not noticed” (23.5%), “App icon too inconspicuous” (5.9%), and “No reminder for the activity button and relaxation modules” (5.9%) were cited as dropout reasons. The app questionnaires should be short and require only a few minutes to complete to address the lack of time for app use, mentioned as a reason for non-participation and dropout by 21.7% of persons in the non-participation group and the dropout group combined. This was already identified in previous research as an important reason not participating in a mental health project in a sample of children and young adults (48). The time factor also emerged in the finding that 17.2% of non-participants reported not having time to install the app. Noting that giving this reason for non-participation, instead of other possible reasons listed in the non-participation questionnaire, could be interpreted as a socially acceptable explanation for non-participation. More appointments should still be offered to install the app to increase participation rates, for example during the next regular therapy appointment.

Another important reason, especially for dropout, concerned the app itself, particularly issues such as disliking the content or layout or unmet expectations. These were reflected in app-related reasons given for dropout, for example “Contents are not individually suitable” (29.4%), “Push messages are annoying” (17.6%) or “Layout not liked” (17.6%). In this context, it is notable that the app was not tailored to one mental disorder but was offered to all respondents regardless of their specific diagnosis of a mental disorder. This possibly made it more difficult to create contents and layouts that meet individual needs, but takes into account that individuals affected by mental disorders are often diagnosed with a combination of mental disorders (5, 6). Engagement of users may be improved by content personalization (30, 31, 47). To address comorbidity, a feasible solution might involve creating an app where, within a certain framework defined by a physician or psychotherapist, users or their treating professionals can activate content tailored to their specific diagnoses. To prevent app-related reasons for dropout and non-participation, involving focus groups and an informed participation of several user groups in the design phase of an app is a promising approach to understand the needs of users (49–51). The focus group session in this study could only be conducted online in response to pandemic restrictions. In future studies, the design and development phase of apps should include more focus group sessions with a larger and more diverse group of participants, along with a stronger emphasis on co-design and a rigorous co-creation process. Involving mental health professionals implementing the apps could help ensure the app aligns with users’ needs (50, 51).

Although research interest in apps has increased in recent years regarding their use in the treatment of mental disorders (19), and clinical benefits have been demonstrated, especially in the area of depressive or anxiety disorders (12), not all patients are willing to use apps. This was evident in our study through reasons for non-participation such as “No interest in using an app for the presented content” (20.7%) and “No interest in an app-based support service” (6.9%), as well as dropout reasons like “Use of face-to-face offerings” (11.8%). Suggesting that despite all trends toward greater use of digital services, traditional in-person offerings remain essential. Nevertheless, a combination of in-person therapy and technology, known as blended care, may positively influence mental health care, for example by enabling broader, possibly more intensive treatment compared to face-to-face therapy alone (52). For instance, presenting the same content in the app as in on-site therapy sessions could allow patients to repeat it at home or to catch up on missed on-site appointments. Studies show that symptom reduction in several mental disorders can be significantly improved by adding app-based interventions to ongoing therapy (53).

Finally, personal health seems to be a relevant factor for non-participation and dropout in this study, with severe health complaints cited by 19.6% of individuals from the non-participation group and the dropout group combined. Among other health complaints such as infections with the coronavirus or an emergency surgery, this included mental health complaints. Notably, this study was conducted in a psychiatric outpatient department of a university hospital, where patients with severe mental disorders receive treatment. Future studies should therefore focus on investigating a cut-off value for improved mental health symptoms after a phase of severe mental health complaints, beyond which app-based support services become appropriate. As a stepped care approach has been shown to be at least as effective or even more effective than usual care in the treatment of several different mental disorders (54, 55), it can be helpful to offer more intensive therapeutic support in a phase of severe mental health complaints. The graphically represented better daily mean mood levels of dropouts compared descriptively to completers during the first twelve days of the app intervention period could be interpreted as meaning that dropouts with more severe mental health complaints dropped out earlier and their mood data were no longer included in the representation of the daily mean mood levels.

When analyzing group differences between dropouts and completers, dropouts reported their mood significantly less often than completers during the first seven days as well as during the first five days of the app intervention period. This is a very important finding, as it may allow potential dropouts to be identified in the early days of a study based on the number of mood reports. Researchers could use this information to contact potential dropouts and investigate the specific reason for their reduced entries in the app. If technical issues are identified as the cause, providing immediate technical support could prevent dropout. Further research is needed to confirm these findings and to evaluate the quantitative difference in the number of entries in the app between potential dropouts and potential completers to establish a predictive indicator of dropout risk.

No other statistically significant differences between dropouts and completers regarding sociodemographic, clinical, and app-related variables were detected, and the initial study hypotheses could not be confirmed. This contrasts with findings from previous literature, which identified sociodemographic, clinical, or app-related parameters associated with dropout (28–31). A possible explanation for this discrepancy may lie in the small sample size of our study, suggesting that future studies on differences between dropouts and completers should be conducted with larger sample sizes. Technical problems have been reported as a reason for lower study adherence in other studies (30, 31), and were not reported by any participant in our study within the first week after study inclusion. This finding could be explained by the high feasibility, acceptance as well as user satisfaction of the app in this study (27).

None of the non-participants indicated “Not liking the content presented” and “Lack of financial compensation” as a reason for non-participation. This suggests that content promoting daily structure, mindfulness, and relaxation is appealing, even without financial compensation. Of all persons declining to participate in this study, only 6.5% refused to indicate the reason for their non-participation. This may be attributable to the design of the non-participation questionnaire, which was deliberately kept short and anonymous to avoid overwhelming non-participants with too many items or a long processing duration. This strategy appeared to be successful with a sample of individuals declining to participate and possibly taking a rejection stance toward the study, which is also supported by the fact that, on average, non-participants reported more than one reason for non-participation. In future studies, it might be useful to collect additional information, such as sociodemographic or clinical data of non-participants to obtain robust factors related to non-participation, possibly with a lower response rate. Reasons for dropout were collected from all dropouts, suggesting that telephone interviews are a useful tool for collecting dropout information. The direct telephone contact between researcher and study participant, coupled with the opportunity for clarification, enabled the collection of detailed and contextualized information about dropout reasons.

Although the results of our study can most likely be transferred to post-pandemic conditions without significant problems, it is important to acknowledge that the study was conducted during COVID-19 pandemic. As a result of governmental restrictions, which were in effect throughout the entire duration of the study, outpatient face-to-face group therapies, for example, could only be conducted with a limited number of patients. That was an exceptional situation for both patients and therapists and may have influenced the results of our study, as both the study participants in the dropout and the completer group stated that they perceived the COVID-19 pandemic to be somewhat psychological distressing. Our findings can be substantiated by the results of a study carried out in the early phase of COVID-19 pandemic, revealing that persons with self-reported anxiety or affective conditions experienced a more acute response and increased stress levels (56). This could also be reflected in our results regarding personal health, as severe health complaints were reported by 19.6% of non-participants and dropouts combined as a reason for non-participation or dropout. It is conceivable that, in the absence of pandemic-related circumstances, health complaints would have been less pronounced, potentially resulting in higher participation rates or lower dropout rates. In our study, 29.4% of dropouts and 37.5% of completers reported suicidal ideation, with suicidal ideation occurring on average more than half of the days in both groups. However, this can most likely be seen in the context that our study was conducted in a psychiatric outpatient department of a university hospital, where patients with severe mental disorders receive treatment. Supporting this interpretation, no increase in suicide rates was observed in the first few months of the COVID-19 pandemic, as a study in several high- and upper-middle-income countries worldwide showed (57). Although 21.7% of persons from the non-participation group and the dropout group combined indicated no motivation to use the app regularly as a reason for non-participation or dropout, the motivation to use alternatives to traditional face-to-face therapy may be affected by the COVID-19 pandemic. In this context, it is conceivable that the motivation to use available alternatives, such as apps, is higher, as this enables uninterrupted treatment against the backdrop of restrictions on on-site therapies in response to governmental restrictions.

### 4.2 Strengths and limitations

A strength of this study is the combination of qualitative and quantitative data, leading to a broader understanding of non-participation and dropout. Further, reasons for non-participation and dropout were assessed in patients with a wide range of diagnoses, avoiding limitations to specific mental disorders and providing a more comprehensive view in the field of mental disorders. In addition, the investigation of non-participation and dropout was conducted among patients receiving treatment in a psychiatric outpatient department of a university hospital, where patients with severe mental disorders receive treatment. To encourage spontaneous and honest responses, the non-participation questionnaire was deliberately kept short and anonymous. Additionally, both non-participants and dropouts were informed that their decision not to participate in the study and their responses in the non-participation questionnaire, as well as early study dropout and any information during the dropout interviews would have no impact on their treatment in the psychiatric outpatient department. Dropout reasons were collected through semi-structured telephone interviews, allowing direct telephone contact between researcher and study participant. This method facilitated a more detailed survey compared to anonymous paper-and-pencil surveys, by enabling clarifications and contextualization of responses. Social desirability was mitigated in the semi-structured telephone interviews by employing open ended questions to avoid implicit suggestions or expectations, maintaining a neutrality in interviewer responses, and ensuring pseudonymized and confidential data handling. Further, the physical distance inherent in the telephone format further contributed. A further strength of this study includes the use of an EMA approach to assess mood data. In this context, the EMA approach is useful because limitations of conventional clinical assessment, such as recall bias, can be reduced by EMA and the validity of the acquired information can be increased (16).

Limitations of this study include the small sample size and the fact that the study was conducted in only one psychiatric outpatient department of one German university hospital. However, the special aspect of our study was that the app was not limited to one specific mental disorder or diagnosis, but could be used by all patients in the psychiatric outpatient department, regardless of the specific diagnosis. To our knowledge, this is the first study to explicitly investigate this in a psychiatric outpatient department of a university hospital. Another limitation is the underrepresentation of male participants, as generally fewer men participated in the study. This gender imbalance affects both the generalizability as well as the interpretability of the study findings. The fact that the majority of study participants are female is in line with previous findings of a study of mental disorders in the general population (58). Because the data of non-participants were collected anonymously, no additional information, such as sociodemographic variables of non-participants, was available. In addition, the reasons for non-participation using an anonymous paper-and-pencil questionnaire and dropout using short protocols of telephone interviews were only investigated using quantitative methods and descriptive statistics. In future studies, mixed methods should be used, telephone interviews should be examined using qualitative methods based on transcripts of the whole interviews, and semi-structured interviews could also be conducted with non-participants to get more details about their non-participation.

### 4.3 Conclusion

Data on dropout and, especially, non-participation remain scarce in studies on smartphone apps in the field of mental disorders. The results of this study provide exclusive insights into reasons why adult outpatients with a mental disorder choose not to participate in or drop out of a study involving an app. These findings have the potential to positively influence the planning of future studies on apps. Personal motivation, app-related aspects, no interest in app-based offers, and personal health complaints were identified as common reasons for non-participation and dropout. Based on these findings, several suggestions for improvement in future studies were developed. Specifically, it could be helpful to create more incentives to use the app to increase participation rates and reduce dropout rates, for example by involving personalized feedback or by adding gamification elements or more push notifications (30, 31, 47). Another way to reduce dropout and increase study participation might be by offering short and quick-to-complete app questionnaires and multiple appointments to install the app. Further, for customizing apps to users’ needs, focus groups and an informed participation of several user groups should be involved in the design phase of apps as much as possible (49, 50). A cut-off value for improved mental health symptoms after a phase of severe mental health complaints could be helpful, beyond which the use of an app-based support service becomes appropriate. Persons who are not interested in app-based offers could also benefit from supporting face-to-face offers by presenting the same content in the app for repetition or to catch up on missed contents of face-to-face appointments in the app.

Another key finding of this study concerns group differences between dropouts and completers. Reports of daily mood were observed significantly less often in the dropout group within the first days of the app intervention period, which could be helpful in future studies to detect potential dropouts at an early stage.

## Data Availability

The raw data supporting the conclusions of this article will be made available by the authors, without undue reservation.
